# Supplementary material for: Inhibition of Eukaryotic Translation Initiation Factor 5A (eIF5A) Hypusination Suppress p53 Translation and Alters the Association of eIF5A to the Ribosomes
Source: Int J Mol Sci. 2020 Jun 27;21(13):4583. doi: 10.3390/ijms21134583 (PMC7369855; doi:10.3390/ijms21134583)
Supplement: Supplementary file 1 [file ijms-21-04583-s001.pdf]

Supplementary Materials

Supplementary figure 1

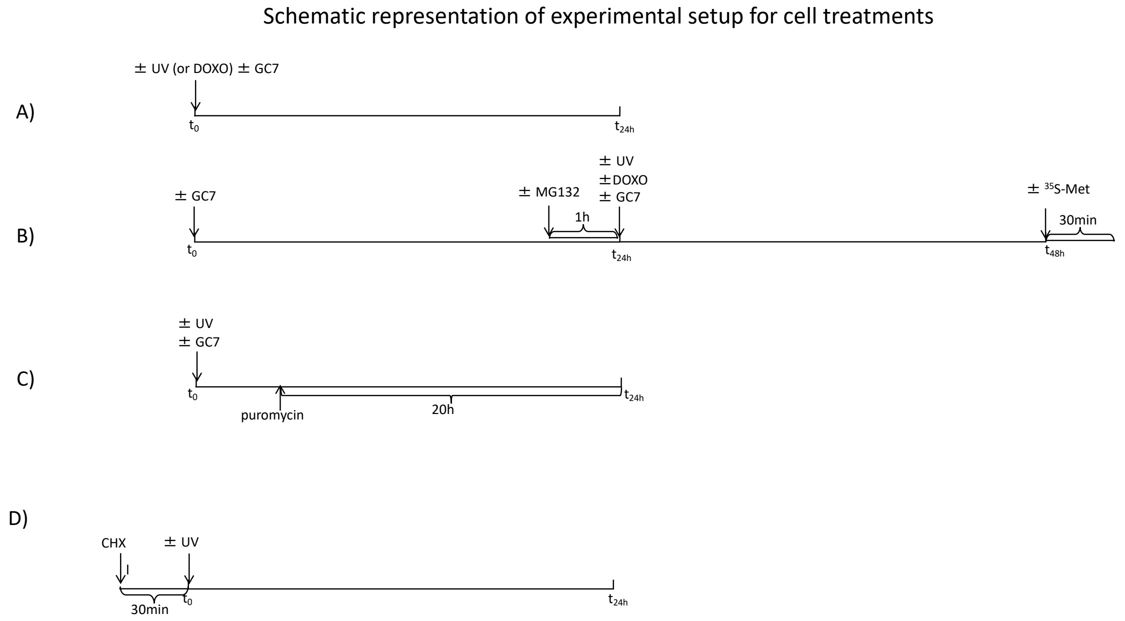

Figure 1S. Schematic representation of different cell treatment

5.2 Supplementary figure 2

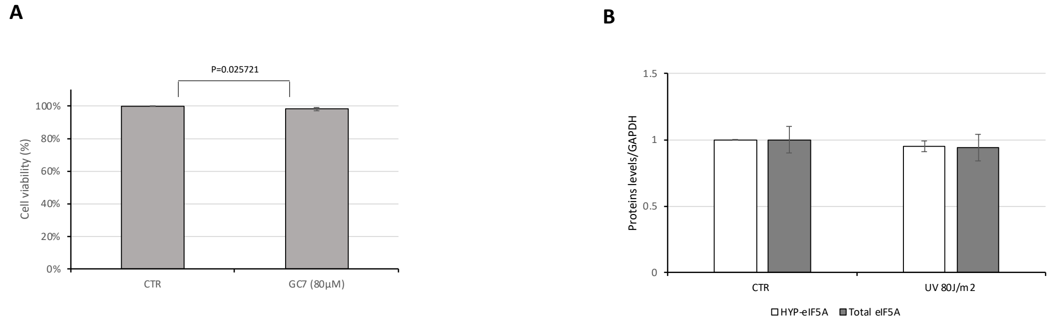

Figure 2S. A) Trypan blue cell viability assay. After treatment with GC7 (80 μM), cell viability was analyzed after 48h of treatment using trypan blue assay. The bar graph shows that GC7 does not affetct cell viability, results obtained from three independent experiments. **B**) Total eIF5A and hyp-eIF5a variations in HCT-116 colon cancer cells untreated and UV treated. The HCT-116 cells were treated with UV-C (80 J/m2) at 24h. Expression of total eIF5A and eIF5A hypusinated form were detected by western blot analysis. Band density was normalized to control GAPDH.

5.3 Supplementary figure 3

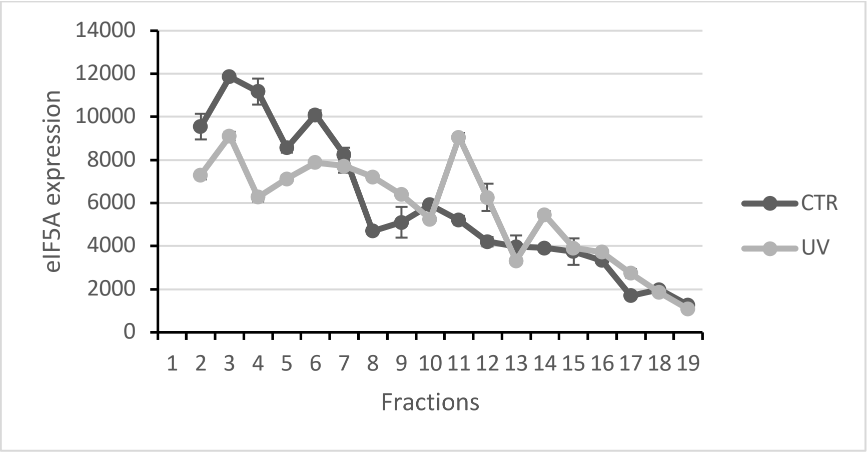

Figure 3S. Quantifications of eIF5A found in the polysome fractions were performed and the graphs are shown the protein distribution.
